# Effect of nicotine on exocytotic pancreatic secretory response: role of calcium signaling

**DOI:** 10.1186/1617-9625-11-1

**Published:** 2013-01-18

**Authors:** Parimal Chowdhury, Kodetthoor B Udupa

**Affiliations:** 1 Department of Physiology & Biophysics, University of Arkansas for Medical Sciences, College of Medicine, 4301 W Markham Street, Little Rock, 72205, Arkansas; 2 Formerly at Donald W. Reynolds Department of Geriatrics, University of Arkansas for Medical sciences, College of Medicine, 4301 W Markham Street, Little Rock, 72205, Arkansas; 3 Formerly in Medical Research, Central Arkansas Veterans Health Care System, Little Rock, Arkansas

**Keywords:** Isolated pancreatic acinar cell, Nicotine, Receptor blocker, Calcium channel antagonists, MAPK kinase inhibitors

## Abstract

**Background:**

Nicotine is a risk factor for pancreatitis resulting in loss of pancreatic enzyme secretion. The aim of this study was to evaluate the mechanisms of nicotine-induced secretory response measured in primary pancreatic acinar cells isolated from Male Sprague Dawley rats. The study examines the role of calcium signaling in the mechanism of the enhanced secretory response observed with nicotine exposure.

**Methods:**

Isolated and purified pancreatic acinar cells were subjected to a nicotine exposure at a dose of 100 μM for 6 minutes and then stimulated with cholecystokinin (CCK) for 30 min. The cell’s secretory response was measured by the percent of amylase released from the cells in the incubation medium Calcium receptor antagonists, inositol trisphosphate (IP_3_) receptor blockers, mitogen activated protein kinase inhibitors and specific nicotinic receptor antagonists were used to confirm the involvement of calcium in this process.

**Results:**

Nicotine exposure induced enhanced secretory response in primary cells. These responses remained unaffected by mitogen activated protein kinases (MAPK’s) inhibitors. The effects, however, have been completely abolished by nicotinic receptor antagonist, calcium channel receptor antagonists and inositol trisphosphate (IP_3_) receptor blockers.

**Conclusions:**

The data suggest that calcium activated events regulating the exocytotic secretion are affected by nicotine as shown by enhanced functional response which is inhibited by specific antagonists… The results implicate the role of nicotine in the mobilization of both intra- and extracellular calcium in the regulation of stimulus-secretory response of enzyme secretion in this cell system. We conclude that nicotine plays an important role in promoting enhanced calcium levels inside the acinar cell.

## Introduction

Smoking is an independent risk factor for development of chronic pancreatitis and pancreatic cancer [1-6]. Studies also show that nicotine plays a significant role in the induction of pancreatic pathophysiology [7-12]. The mechanism of this effect of nicotine on the pancreas remains elusive and has not been established yet. We have designed the current study in freshly isolated pancreatic acinar cells to determine the direct effect of nicotine on exocrine secretory capacity by manipulating calcium selective agents and mitogen activated protein kinase (MAPK) inhibitors since in-vivo studies have shown that nicotine at pharmacological concentrations decrease exocrine function via suppression of amylase release [7,11,13-15]. It is suspected that these effects are most likely mediated via calcium regulated pathways [9,16,17]. However, no confirmatory studies have been reported. In the current study we confirm that these effects are regulated by calcium signaling as calcium-selective inhibitors suppressed the nicotine-induced pancreatic secretory response. The rationale for the selection of various calcium sensing antagonists for the study has been briefly described. Our major hypothesis is that while calcium is important in regulating the normal exocytotic secretory processing, excess intracellular calcium that is produced with nicotine exposure at its pharmacological doses may suppress pancreatic function leading to inflammation [7-9].

While Ca^2+^ could be released from the [Ca^2+^_I_ stores including store –operated calcium channels, activation of inositol-1,4,5-trisphosphate (IP_3_) receptors, found at the cellular membrane, results in an elevation of [Ca^2+^_i_[18]. 2-Aminoethoxydiphenyl borate (2-APB) is a reliable blocker of store-operated Ca^2+^ entry as well as an inhibitor of InsP_3_-induced Ca^2+^ release [19]. α-conotoxins are tightly folded miniproteins that antagonize nicotinic acetylcholine receptors (nAChR) with high specificity for diverse subtypes. ω-conotoxin inhibits N-type voltage-dependent calcium channels [20]. H-7 dihydrochloride is a broad-based, cell-permeable serine/threonine kinase inhibitor and has often been used to assess the contribution to cellular processes, including the induction of gene expression. H-7 inhibits PKC more potently in *in-vitro* assays and is mainly used as a rather nonspecific inhibitor of protein kinase activity [21]. Mecamylamine (inversine) is a nonselective and noncompetitive antagonist of the nicotinic acetylcholine receptors and it blocks the effect of nicotine [22,23].

## Material and methods

### Reagents

All reagents used in the experiment are of analytical grade. Nicotine was purchased from Sigma (St. Louis, MO) and it was obtained in liquid form. Nicotine was dissolved initially with a few drops of ethanol and further diluted to the required concentration with saline, pH adjusted to 7.4 by sodium hydroxide (1M). For control samples, medium containing the same amount of ethanol was used as was done for dissolution of nicotine with saline, the pH adjusted to 7.4. Cholecystokinin (CCK-8) was purchased from Bachem, Philadelphia, PA. For inhibitor studies, MAPK inhibitor, UO126, jun-kinase inhibitor and p-38 kinase inhibitors were purchased from (Cell Signaling Technology, Inc., Beverly, MA). 2-Aminoethoxydiphenyl borate (2-APB), a reliable blocker of store-operated Ca^2+^ entry and H-7, a broad-based, cell-permeable serine/threonine kinase inhibitor, were purchased from Calbiochem (San Diego, California). Mecamylamine, a nicotinic acetylcholine receptor antagonist, was purchased from Sigma Life Sciences (St. Louis, MO). ω-conotoxin, an N-type voltage-dependent calcium channels inhibitor was purchased from Peptide International (Louisville, Kentucky).

#### Isolation of primary pancreatic acinar cells

Adult male Sprague Dawley rats were used for the study. The animals were procured through a protocol approved by the Institutional Animal Care and Use Committee. The animals were acclimatized for a week under controlled laboratory conditions prior to the study. After an 18-hour fast, the animals were sacrificed, the pancreas removed quickly and freed from fat and lymph nodes. Pancreatic acini were isolated by enzymatic digestion according to methods reported previously [17,24,25]. Briefly, Krebs-Henseleit bicarbonate buffer, pH 7.4 (KHB), containing the minimum Eagle’s Medium supplement (MEM), 67 U/ml collagenase, 2 mg/ml bovine serum albumin (BSA), and 0.1 mg/ml soybean trypsin inhibitor, was injected into the pancreatic tissue interstitium. The injected pancreatic tissue was incubated at 37°C in a shaking water bath at a frequency of 120 times/min for 40 minutes, followed by mechanical disruption of the tissue with gentle suction through pipettes of decreasing orifice sizes. Acini were then purified by filtration through 150 μM polyethylene mesh and by density gradient centrifugation with KHB containing 4% BSA. Acini were preincubated for 30 minutes in HEPES-buffered Ringer’s solution, pH 7.4 (HR). The HR used was the same as KHB, except that it contained 10 mmol/L Hepes and 0.5% BSA. Prior to use, the buffer was gassed with 100% O_2_. After pre-incubation, acini were washed and resuspended in fresh HR at a density of 0.3-0.4 mg/ml of acinar protein.

#### Primary cell culture

The purified primary acinar cells were maintained overnight in 100 mm culture dish at concentration of 1.6 X 10^6^/10 ml in culture media containing Ham’s F-12 nutrient medium (F12K) with 2 mM L-glutamine, 1% antibiotic, 1.5% sodium bicarbonate, and 10% fetal bovine serum albumin (FBS) at 37°C in a 5% CO_2_/95% air atmosphere. On day one, the medium was changed to a serum-free nutrient medium.

#### Treatment of cells with nicotine

The cells were treated with 10 μM to 500 μM of nicotine (Sigma, St. Louis, MO) in 6.0 ml of serum-free medium for periods of 30 seconds to 10 minutes. Cells were pretreated with inhibitors for 30 minutes before adding nicotine.

#### Amylase secretion from primary cells after nicotine treatment

Cell function studies were performed with or without CCK (Bachem, Philadelphia, PA) at its maximal, previously determined stimulating dose. These studies were conducted with primary cells that were washed free of media with Hepes-Ringer (HR) buffer, pH 7.4 (2X), incubated in the same buffer with or without nicotine (50 μM to 1000 μM) for periods of 0 to 10 minutes and then washed twice with HR buffer to make the cells nicotine-free. The cells were then dispersed in fresh HR buffer, incubated with or without CCK (10^-9^ M) for 30 minutes at 37°C. The selection of this dose of CCK (10^-9^ M) for was based on our initial study, which showed a maximal stimulated response of amylase release in a CCK dose–response curve [14]. Following the incubation period, the media was removed by centrifugation and analyzed for amylase activity by the method of [26] with Procion yellow starch as substrate (PRO Chemical & Dye, Somerset, MA). Cell pellets were washed with ice-cold PBS, lysed with water by sonication, and centrifuged. The cell lysate was analyzed for both amylase and protein content. Protein concentration was measured by the method of [27]. The amylase release was expressed as the fractional amount (%) released from the total.

#### Effect of mecamylamine or conotoxin on primary cell function, with or without nicotine

Primary cells (4–6 x 10^6^) were washed and incubated at 37°C with or without 500 μM mecamylamine for 30 minutes, followed by an additional incubation with nicotine for 6 minutes. Cells were washed and then incubated at 37°C with or without CCK-8 (10^-9^ M) for 30 minutes. Amylase released into the incubation medium was measured with Procion-yellow starch as substrate. The data are presented as % initial content and represented as the mean ± SEM of four experiments.

For conotoxin experiment, primary cells (4–6 x 10^6^) were washed and incubated at 37°C without or with 80 mM conotoxin for 30 minutes, followed by additional 6 minutes incubation with nicotine. The rest of the procedure was similar as described above.

#### Effect of H-7 on primary cell function, with or without nicotine

Primary cells (4–6 x 10^6^) were washed and incubated at 37°C with or without 10 μM H-7 for 30 minutes, followed by an additional incubation with nicotine for 6 minutes. Cells were washed and then incubated at 37°C with or without CCK-8 (10^-9^ M) for 30 minutes. Amylase released into the incubation medium was measured with Procion-yellow starch as substrate. The data are presented as % initial content and represented as the mean ± SEM of four experiments.

#### Effect of IP_3_ receptor antagonist on acinar cell function with or without nicotine

Primary cells (4–6 x 10^6^) were washed and incubated at 37°C without or with 80 mM 2-APB for 30 minutes, followed by an additional 6 minutes incubation with nicotine. Amylase released into the incubation medium was measured with Procion-yellow starch as substrate. The data are presented as % initial content and represented as the mean ± SEM of four experiments.

#### Effect of ERK Inhibitor on primary cell function with or without nicotine

To study the effect of an ERK1/2 inhibitor on basal and stimulated amylase release, primary cells were pretreated with UO126 (10 μM) for 30 minutes. After washing, the cells were incubated with 100 μM nicotine for 6 minutes. At the end of the incubation period, the cells were washed with HR buffer, and incubated with either buffer, or buffer containing 10^-9^ M CCK-8, for 30 minutes. Amylase release in the media and protein concentration were determined as outlined in the earlier section. The method used for CCK stimulation was similar to that described in the earlier section.

#### Effect of JNK inhibitor or p38 inhibitor on primary cell function, with or without nicotine

Primary cells (4–6 x 10^6^) were washed and incubated at 37°C with or without 100 nM JNK inhibitor or with 1 μM p38 kinase inhibitor for 30 minutes, followed by an additional incubation with nicotine for 6 minutes. Cells were washed and then incubated at 37°C with or without CCK-8 (10^-9^ M) for 30 minutes. Amylase released into the incubation medium was measured with Procion-yellow starch as substrate. The data are presented as % initial content and represented as the mean ± SEM of four experiments.

#### Statistics

Results were reported as mean ± standard error of the mean (SEM). The results were analyzed by student’s *t*-test and where required, with analysis of variance (ANOVA). Differences were considered significant at a probability of 0.05 or lower.

## Results

### Nicotine Effects on CCK-stimulated acinar cell function

**Figure 1:**
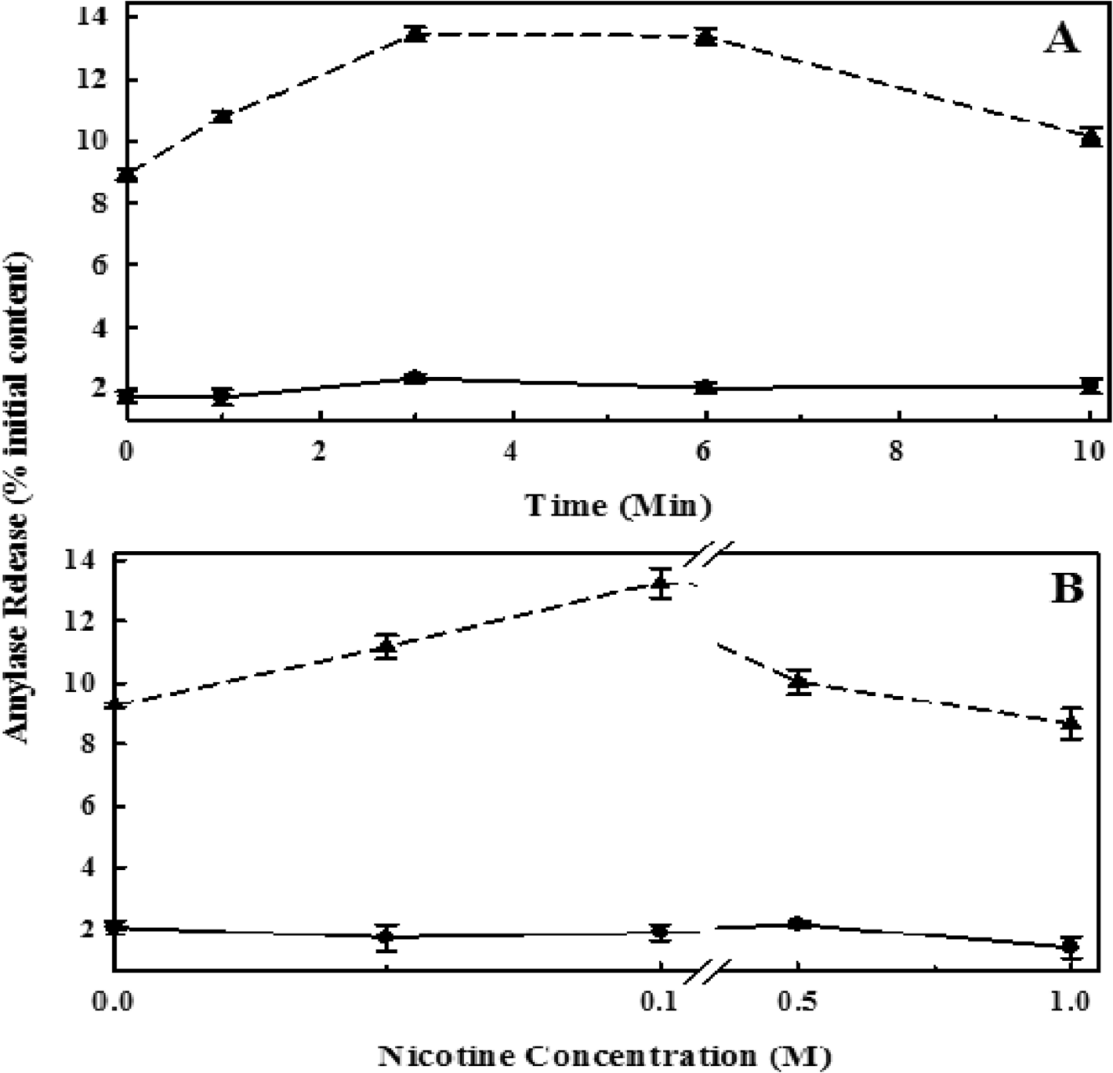
**Primary cells were washed, incubated at 37°C with 100 μM nicotine for 0 to 10 minutes (Panel A) or at various concentrations of nicotine (Panel B) and then incubated with CCK-8 (10**^**-9**^**) M, or without CCK-8 for 30 minutes.** Amylase released into the medium was measured with Procion-yellow starch as substrate. The data are presented as % initial content and represented as the mean ± SEM of four separate experiments (unstimulated, circles with solid line); (stimulated, triangles with dashed line).

The time and nicotine dose effects on maximal stimulated amylase release in response to CCK are shown in Figure 1. The solid line represents the amylase response of control cells while the broken line represents the stimulated amylase response by CCK at a maximal stimulating dose of 10^-9^ M [14]. The bottom panel in Figure 1 shows that nicotine at a dose of 100 uM induced maximal release of amylase (Panel B) while the upper panel (Panel A) shows that the maximal response occurring at 3 mins of exposure that persisted until 6 min before decline. Based on this observation, a single dose of nicotine (100 uM) and time of exposure for 6 min was used in subsequent experiments on the results as shown below.

### Effect of mecamylamine or conotoxin on primary cell function, with or without nicotine

**Figure 2:**
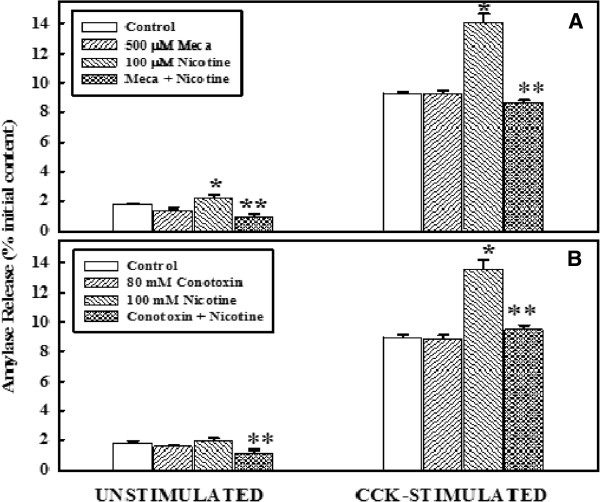
**Effect of mecamylamine or conotoxin on primary cell function, with or without nicotine. Panel A**: Primary cells (4–6 x 10^6^) were washed and incubated at 37°C with or without 500 μM mecamylamine for 30 minutes, followed by an additional incubation with nicotine for 6 minutes. Cells were washed and then incubated at 37°C with or without CCK-8 (10^-9^ M) for 30 minutes. Amylase released into the incubation medium was measured with Procion-yellow starch as substrate. The data are presented as % initial content and represented as the mean ± SEM of four experiments. **Panel B**: Primary cells (4–6 x 10^6^) were washed and incubated at 37°C without or with 80 mM conotoxin for 30 minutes, followed by additional 6 minutes incubation with nicotine. The rest of the procedure was similar to panel A. The data are presented as % initial content and represented as the mean ± SEM of four experiments. *P < .05 between control and nicotine-added samples; **P < .05 between nicotine-added and mecamylamine + nicotine-added or conotoxin + nicotine added samples.

The effects of nicotine in the presence or absence of mecmylamine, and conotoxin on the basal and cell stimulated primary acinar cell function are shown in Figure 2. The upper panel (panel A) represents the basal and stimulated amylase release in the presence of mecamylamine while the lower panel (panel B) represents the effect of conotoxin. As shown in panel A, the basal levels of amylase release was increased significantly in the presence of nicotine when compared to control levels and reduced significantly in the presence of mecamylamine. Stimulation with CCK-8 enhanced the levels of amylase release in all groups. Treatment with nicotine induced CCK stimulated levels more than the control group. However, in the presence of mecamylamine, the enhanced response induced by nicotine was completely abolished returning the stimulated levels to that of control level. The response of acinar cell to amylase release by nicotine in the presence of conotoxin is identical to that of mecamylamine (Panel B).

### Effect of H-7 or 2-APB on primary cell function, with or without nicotine

**Figure 3:**
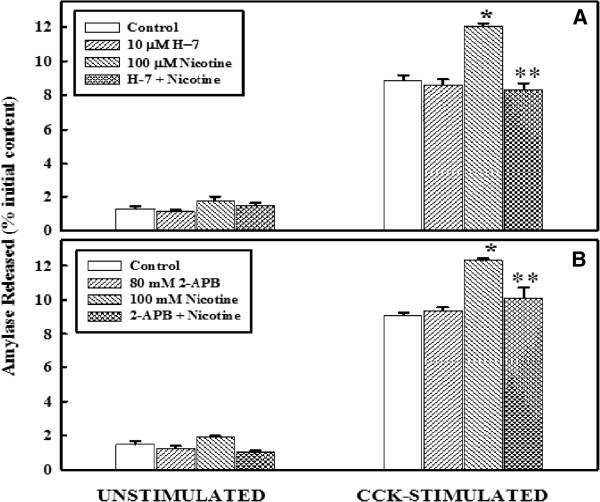
**Effect of H-7 or 2-APB on primary cell function, with or without nicotine. Panel A**: Primary cells (4–6 x 10^6^) were washed and incubated at 37°C with or without 10 μM H-7 for 30 minutes, followed by an additional incubation with nicotine for 6 minutes. Cells were washed and then incubated at 37°C with or without CCK-8 (10^-9^ M) for 30 minutes. Amylase released into the incubation medium was measured with Procion-yellow starch as substrate. The data are presented as % initial content and represented as the mean ± SEM of four experiments. **Panel B**: Primary cells (4–6 x 10^6^) were washed and incubated at 37°C without or with 80 mM 2-APB for 30 minutes, followed by an additional 6 minutes incubation with nicotine. Amylase released into the incubation medium was measured with Procion-yellow starch as substrate. The data are presented as % initial content and represented as the mean ± SEM of four experiments. *P < .05 between control and nicotine-added samples; **P < .05 between nicotine-added and H-7 + nicotine-added or 2-APB + nicotine added samples.

The effects of nicotine in the presence or absence of H-7, and 2-APB on the basal and cell stimulated primary acinar cell function are shown in Figure 3. The upper panel (panel A) represents the basal and stimulated amylase release in the presence of H-7 while the lower panel (panel B) represents the effect of 2-APB. As shown in panel A, the basal levels of amylase release were similar in all four groups. Stimulation with CCK-8 enhanced the levels of amylase release in all groups. Treatment with nicotine induced CCK stimulated levels more than the control group. However, in the presence of H-7, or 2-APB the enhanced response induced by nicotine was completely abolished and returned to stimulated value as of control. The response of acinar cell to amylase release by nicotine in the presence of H-7 (Panel A) is identical to that of 2-APB (Panel B).

### Effect of ERK and AKT Inhibitor on primary cell function with or without nicotine

**Figure 4:**
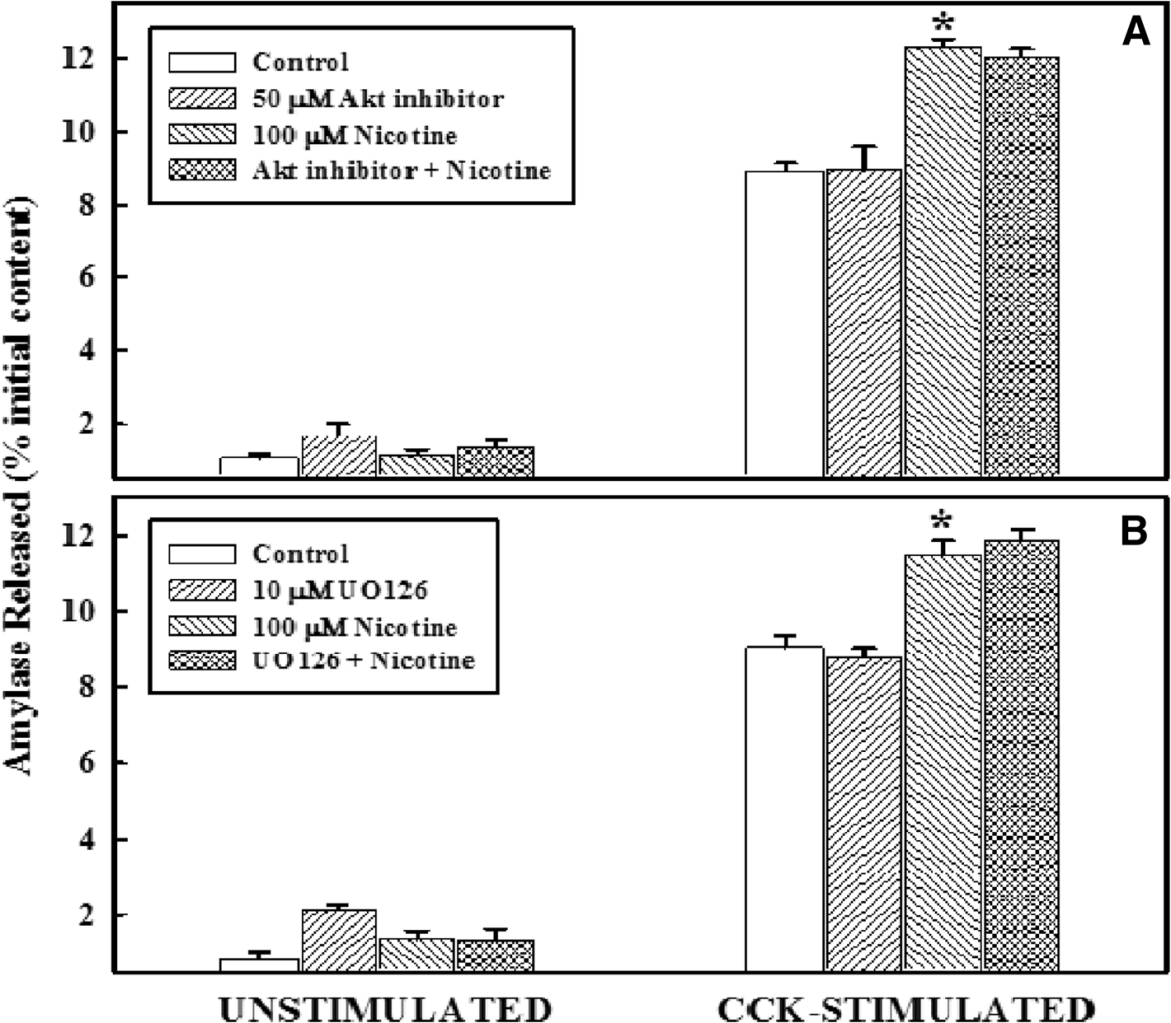
**Effect of AKT inhibitor or ERK1/2 inhibitor on primary cell function, with or without nicotine. Panel A**: Primary cells (4–6 x 10^6^) were washed and incubated at 37°C with or without 50 μM AKT inhibitor for 30 minutes, followed by an additional incubation with nicotine for 6 minutes. Cells were washed and then incubated at 37°C with or without CCK-8 (10^-9^ M) for 30 minutes. Amylase released into the incubation medium was measured with Procion-yellow starch as substrate. The data are presented as % initial content and represented as the mean ± SEM of four experiments. **Panel B**: Primary cells (4–6 x 10^6^) were washed and incubated at 37°C without or with 10 μM UO126 for 30 minutes, followed by an additional 6 minutes incubation with nicotine. Amylase released into the incubation medium was measured with Procion-yellow starch as substrate. The data are presented as % initial content and represented as the mean ± SEM of four experiments. *P < .05 between control and nicotine-added samples.

The effects of nicotine in the presence or absence of MAPK inhibitors, UO126, and AKT on the basal and cell stimulated primary acinar cell function are shown in Figure 4. The upper panel (panel A) represents the basal and stimulated amylase release in the presence of AKT inhibitor while the lower panel (panel B) represents the effect of UO126. As shown in panel A, the basal levels of amylase release were similar in all four groups. Upon stimulation with CCK-8, levels of amylase release were enhanced significantly in all groups in all groups. Treatment with nicotine induced CCK stimulated levels more than the control group. However, in the presence of AKT inhibitor, or UO126, the enhanced response induced by nicotine remained unaltered. The response of acinar cell to amylase release by nicotine in the presence of AKT inhibitor (Panel A) is identical to that of UO126 (Panel B).

### Effect of JNK inhibitor or p38 inhibitor on primary cell function, with or without nicotine

**Figure 5:**
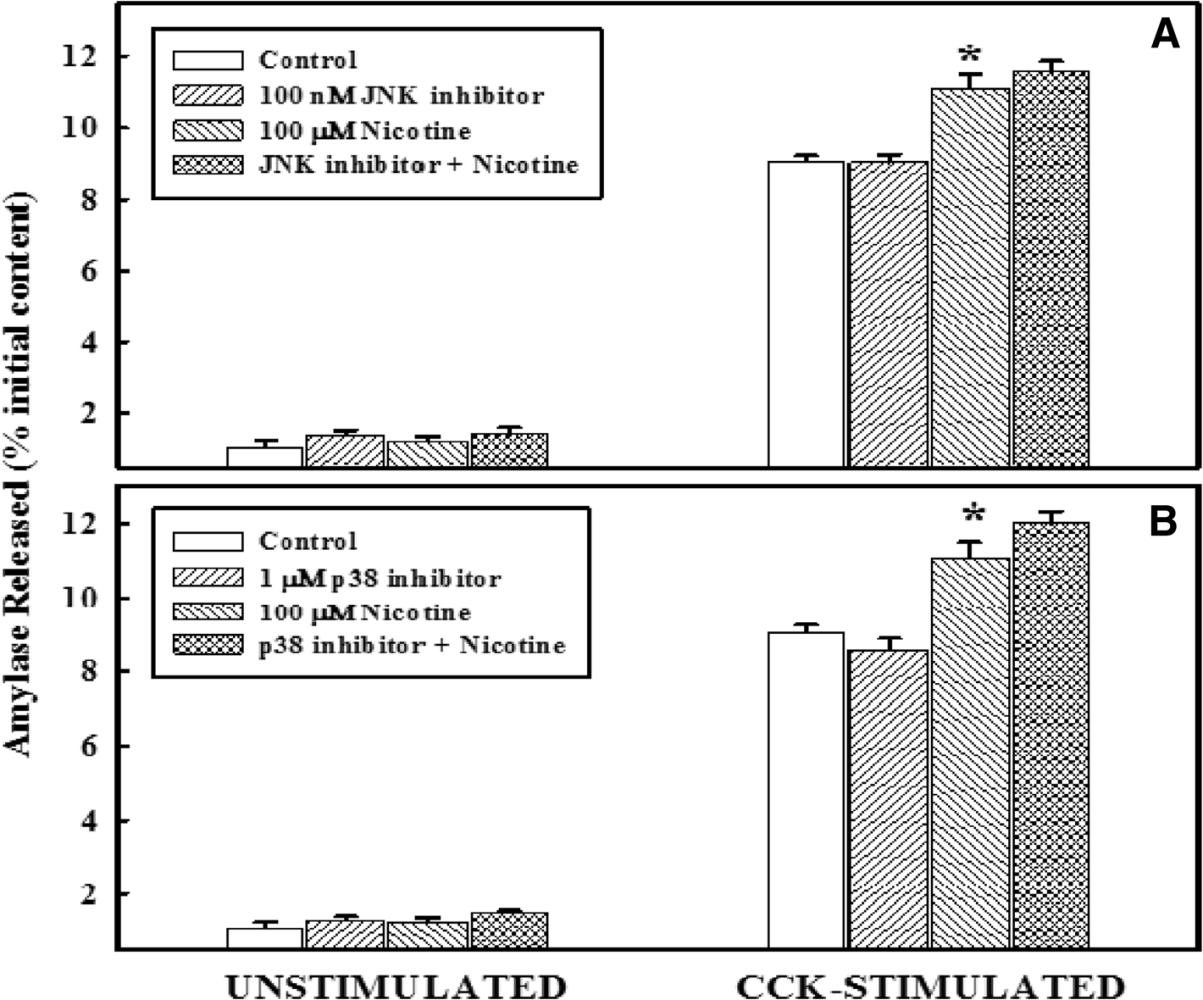
**Effect of JNK inhibitor or p38 inhibitor on primary cell function, with or without nicotine. Panel A**: Primary cells (4–6 x 10^6^) were washed and incubated at 37°C with or without 100 nM JNK inhibitor for 30 minutes, followed by an additional incubation with nicotine for 6 minutes. Cells were washed and then incubated at 37°C with or without CCK-8 (10^-9^ M) for 30 minutes. Amylase released into the incubation medium was measured with Procion-yellow starch as substrate. The data are presented as % initial content and represented as the mean ± SEM of four experiments. **Panel B**: Primary cells (4–6 x 10^6^) were washed and incubated at 37°C without or with 1 μM p38 inhibitor for 30 minutes, followed by an additional 6 minutes incubation with nicotine. Amylase released into the incubation medium was measured with Procion-yellow starch as substrate. The data are presented as % initial content and represented as the mean ± SEM of four experiments. *P < .05 between control and nicotine-added samples.

The effects of nicotine in the presence or absence of MAPK inhibitors, JNK, and p38 kinase inhibitors on the basal and cell stimulated primary acinar cell function are shown in Figure 5. The upper panel (panel A) represents the basal and stimulated amylase release in the presence of JNK inhibitor while the lower panel (panel B) represents the effect of p38 kinase inhibitor. As shown in panel A, the basal levels of amylase release were similar in all four groups. Upon stimulation with CCK-8 levels of amylase release were enhanced significantly in all groupss. Treatment with nicotine induced CCK stimulated levels more than the control group. However, in the presence of JNK inhibitor, or p-38 kinase inhibitor, the enhanced response induced by nicotine was not changed. The response of acinar cell to amylase release by nicotine in the presence of JNK inhibitor (Panel A) is identical to that of p-38 kinase inhibitor (Panel B).

## Discussion

The data presented in this communication demonstrates, in part, the mechanism by which nicotine induced the enhanced secretory response in pancreatic acinar cells

In earlier studies the effect of nicotine on cell signaling and function in this cell system and in rat tumorigenic cell line has been reported from this laboratory [28-30]. However, the precise mechanism by which nicotine induces the enhancement of the acinar cell function was not shown. The initial focus of the current study was to confirm the effects of nicotine on stimulated secretory response in primary cells and then to evaluate its mechanism. As shown in Figures (2–5), nicotine treatment maximally increased basal and CCK-stimulated enzyme secretions at 6 min of nicotine exposure. The dose of nicotine used for the study was identical to that was used in our earlier reported studies [28-30]. The selection of the nicotine dose was based on published literature reported both in in-vivo and other cell culture studies [7,8,31-35]. Dose levels of nicotine used in our study were below peak plasma nicotine concentration found in chronic cigarette smokers, which ranges from 10 to 15 mM, measured within 20 min of cigarette smoking [33]. In addition, in other laboratories, nicotine doses at varying concentration ranging from 0.75 mM to 25 mM have been used in isolated rat pancreatic acini [34]. In our current study, a lower nicotine dose of 100 uM, was used resulting in an induction of maximal secretory response when exposed for 3 min, which persisted for 6 min before decreasing (Figure 1). This is consistent with our findings published earlier.

We have reported earlier that nicotine acts as a mitogen in acinar cell system by activating p-ERK 1 and 2 [28-30,36]. Our studies show that ERK1/2 is activated by nicotine treatment under similar conditions and in the presence of the nicotine receptor antagonist the stimulatory cell response remain unaffected, implying that the kinase and secretory responses induced by nicotine are completely independent of each other and, perhaps, involve a separate mechanism. In this study we have looked into the influence of MAPK activation by nicotine and its effects on cell function. As shown in Figures 4 and 5, mitogen activated protein kinases have no influence on nicotine-induced CCK-stimulated cell function suggesting that response of nicotine on cell function is regulated by a mechanism not related to MAPK activation.

The effects of nicotine on basal and stimulated secretory response are abolished by mecamylamine, a nicotinic receptor antagonist, suggesting that the secretory response of nicotine is receptor-mediated. It has long been established that stimulation of enzyme secretions by secretagogues in pancreatic acini involves several second-messenger pathways that are rapidly activated by G-protein-coupled receptors [37-39] and changes in intracellular calcium concentration [40,41] and may involve a cell-mediated intracellular Ca2+ response [9,42]. In this study, the suppression of the secretory responses by calcium selective antagonists as shown in Figures 2 and 3 indicates that enhanced secretion induced by nicotine is triggered via calcium-activated process. Enhanced release of calcium at pharmacological doses of nicotine may lead to loss of pancreatic function resulting in pancreatic pathology [7-9]. Thus the studies may be clinically relevant to the development of pancreatitis in smokers and will most likely be based on nicotine dose derived from the number of cigarettes smoked.

It has been reported that in rat sublingual mucous acini, nicotine first triggers the release of acetylcholine from pre-synaptic nerve terminals, which then activates muscarinic receptors [43]. In this study, we have observed that w-conotoxin, a potent Q-type calcium channel blocker, completely inhibited nicotine-induced pancreatic secretion suggesting that the regulation of pancreatic secretion by nicotine is physiologically regulated by a calcium-mediated process. The current study has examined the specificity of the nicotinic receptor antagonist in primary cells and has demonstrated that its effect on downstream events regulating exocrine secretion is regulated by calcium activated events involving both intra and extracellular calcium mobilization.

It has been demonstrated that intracellular calcium ([Ca^2+^_i_) signals are involved in a number of events, including apoptotic pathways [44-46]. While Ca^2+^ could be released from the [Ca^2+^_i_ stores, Ca^2+^ also could enter from the extracellular space through membrane channels. Recently it has been shown that the activation of inositol-1, 4, 5-trisphosphate (IP_3_) receptors, found at the cellular membrane, results in an elevation of [Ca^2+^_I_[18]. The modulation of [Ca^2+^_I_ influences the activation of calplain [47], which controls cell-cycle regulation, differentiation and apoptosis [48]. Activation of calpain from pro-calpain leads to apoptosome formation by cleavage of Bid, which in turn regulates Bax and activates caspase-3 [44,48].

## Conclusions

In summary, our data suggest that calcium activated events regulating the exocytotic secretion are affected by nicotine inducing enhanced functional response as confirmed by the inhibitory actions of these specific antagonists… The results implicate the role of nicotine in the mobilization of both intra- and extracellular calcium in the regulation of stimulus-secretory response of enzyme secretion in this cell system. We conclude that nicotine plays an important role in promoting enhanced calcium levels inside the acinar cell.

## Competing interest

The authors have no conflict of interest to declare.

## Authors’ contribution

Authors PC, KU had the main role in study design, data analysis, data interpretation and manuscript preparation while the research Assistant HZ was responsible for data collection, organization, and participated in data Interpretation and manuscript preparation. Both authors read and approved the final manuscript.

## Funding

This work was supported, in part, by a grant to PC from the University of Arkansas for Medical Sciences.
